# Detection of SARS-CoV-2 on surfaces in food retailers in Ontario

**DOI:** 10.1016/j.crfs.2021.08.009

**Published:** 2021-08-31

**Authors:** Maleeka Singh, Azin Sadat, Reihaneh Abdi, Louis A. Colaruotolo, Alyssa Francavilla, Katherine Petker, Pedram Nasr, Maryam Moraveji, Gyllian Cruz, Yinan Huang, Aditi Arora, Aleana Chao, Sarah Walker, Xinya Wang, Sujani Rathnayake, Subramanyam Ragupathy, Steven G. Newmaster, Robert H. Hanner, Lawrence D. Goodridge, Maria G. Corradini

**Affiliations:** a Food Science Department, University of Guelph, Guelph, ON, Canada; b Arrell Food Institute, University of Guelph, Guelph, ON, Canada; c Integrative Biology Department, University of Guelph, Guelph, ON, Canada

**Keywords:** SARS-CoV-2, High-touch surfaces, Food retailers, RT-qPCR, Virus transmission, COVID-19

## Abstract

The COVID-19 pandemic has generated increased interest in potential transmission routes. In food retail settings, transmission from infected customers and workers and customers through surfaces has been deemed plausible. However, limited information exists on the presence and survival of SARS-CoV-2 on surfaces, particularly outside laboratory settings. Therefore, the purpose of this project was to assess the presence of the virus at commonly found surfaces at food retail stores and the potential role that these spaces play in virus transmission.

Samples (n=957) were collected twice a week for a month in food-retail stores within Ontario, Canada. High-touch surfaces were identified and surveyed in 4 zones within the store (payment stations, deli counters, refrigerated food section and carts and baskets). The samples were analyzed using a molecular method, i.e., reverse transcriptase quantitative Polymerase Chain Reaction (RT-qPCR).

Regardless of the store's location, the sampling day or time, the location of the surface within the store or the surface material, all samples tested negative for SARS-CoV-2. These results suggest that the risk of exposure from contaminated high-touch surfaces within a food retailer store is low if preventive measures and recommended sanitizing routines are maintained.

## Graphical abstract

**Figure:**
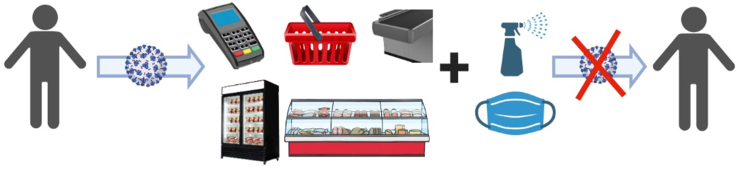

## 1 Introduction

On March 11th, 2020, the World Health Organization declared the coronavirus disease 2019 (COVID-19) a global pandemic (WHO, 2020). As a result, numerous countries decreed State of Emergencies, with lockdowns and travel restrictions implemented to reduce the spread of the virus. Since then, COVID-19, caused by the Severe Acute Respiratory Syndrome Coronavirus 2 (SARS-CoV-2), has infected millions of people worldwide, with fatalities in the hundreds of thousands (Bhardwaj and Agrawal, 2020; JHU, 2021; Li et al., 2020).

To curb SARS-CoV-2 spread until treatment and full vaccination of the population can occur (Aboubakr et al., 2020), it is of utmost importance to identify potential routes and susceptible spaces for transmission. SARS-CoV-2 primarily spreads through direct personal contact, respiratory droplets, and bodily fluids (CDC, 2020; Li et al., 2020; WHO, 2020). Recent evidence suggests that indirect transmission, i.e., becoming infected by touching inanimate objects or surfaces (fomites) that have come into contact with the virus and then touching eyes, nose or mouth, is feasible (Fiorillo et al., 2020). Prior studies have provided information to support the link between virus transmission and contact surfaces (Kampf et al., 2020; Marquès and Domingo, 2021). However, the research has focused on surveying SARS-CoV-2 presence on contact surfaces, assessing its persistence on different materials, and determining its infectivity through time in laboratory settings (Carraturo et al., 2020; Chin et al., 2020). Although limited information is available on tests performed under real-life situations, the presence of SARS-CoV-2 genetic material has been reported on surfaces within environments with high viral loads, such as hospital wards and patients’ rooms (Li et al., 2020; Razzini et al., 2020). The RNA of SARS-CoV-2 has also been detected on door handles, cell phones and other surfaces in residential sites of infected individuals (Aboubakr et al., 2020). Additionally, the transfer of viral RNA of a surrogate from different areas within a facility through touch has been reported (Rawlinson et al., 2020). Viral persistence and the ability to remain active are contingent on numerous factors such as airflow, temperature and relative humidity within an indoor facility. The type of material that the virus is in contact with has also been reported to affect persistence. A recent study found that SARS-CoV-2 was viable for 4 h on copper, 24 h on cardboard, and 72 h on plastic and stainless steel (van Doremalen et al., 2020). Another study reported that the human coronavirus strain HCoV-229E, which is closely related to SARS-CoV-2, could survive on various surfaces such as metal, glass or plastic for 2 h to 9 days, with temperatures of 30 °C or 40 °C reducing viral persistence and survivability (Kampf et al., 2020).

Finally, the presence of SARS-CoV-2 on surfaces will depend on the health of the workers in that space. The link between exposure, infection rate and occupation is evident for health care workers (Reusken et al., 2020). The incidence of the COVID-19 on essential workers in the retail industry, specifically supermarkets, has been reported to be as high as within health care professionals (~20%), and the majority of the workers that tested positive were asymptomatic (Lan et al., 2020; Sim, 2020). The above-cited study focused on the health and extent of transmission among the workers. The high incidence among workers suggests that they could become a source of the virus within the retail stores, and lack of compliance with preventive measures (sanitation routines, use of protective gear, and staying at home while sick) could increase the presence of the virus on fomites.

Taking into account the persistence of SARS-CoV-2 on various surfaces, the relatively low infective dose, and the incidence of the disease in essential workers, indirect transmission from environments other than hospitals should be seriously considered (Duda-Chodak et al., 2020; Warnes et al., 2015; Yekta et al., 2021). Therefore, expanding the research to real-life situations and spaces that, despite the quarantine and lockdown restrictions, are still open, such as food stores, grocery stores, and retailers, should be prioritized to adequately assess the risk of indirect viral transmission in these indoor environments.

Currently, limited information exists on the presence and survival of SARS-CoV-2 on surfaces under everyday conditions (Colaneri et al., 2020a, Colaneri et al., 2020b; Mondelli et al., 2020), and no information in retail food stores has been reported to date. This project aims to assess the presence of SARS-CoV-2 on commonly found surfaces at food retail stores to evaluate the role these spaces may play in this virus transmission.

## 2 Materials and methods

### 2.1 Store selection

Stores were selected based on location. Ontario has been one of the primary provinces in Canada affected by the pandemic, along with Quebec and Alberta, regardless of the COVID-19 wave considered. Although retailers close to the University of Guelph were prioritized to facilitate the logistics in terms of transportation of the samples, a 90 km radius was covered to ensure that food retailers located in urban and suburban areas were included. The size and population density of the surrounding communities was also diverse, ranging from 20,000 to 700,000 inhabitants and 500 to 2500 people per square kilometer, respectively (Statistics Canada, 2017). Therefore, the stores chosen were not isolated, were in population-dense areas, which generated high traffic, and were located in a COVID-19 “hot zone.” The stores selected were in 4 different municipalities/towns within a single province. Hence, the provincial and federal COVID-19 restrictions and guidelines were the same for all. All retail stores tested had in place similar protocols that complied with the provincial requirements (Ontario Ministry of Health, 2020) in terms of social distancing, sanitation, and exposure. All tested retailers also enforced the use of PPE among their patrons (e.g., masks were mandatory) and workers (e.g., masks were mandatory, face shields were available, but its use was voluntary, and gloves were mandatory in food preparation areas and voluntary in non-food preparation areas).

### 2.2 Selection of sampling zones, surfaces, and testing times

**Fig. 1:**
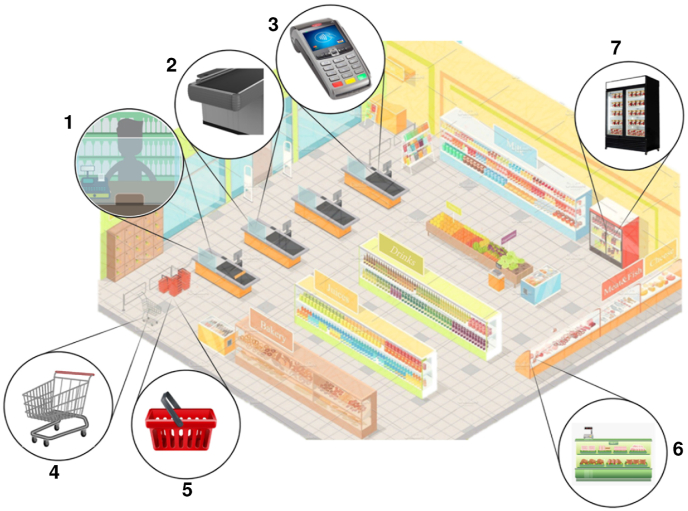
Schematic layout of a retailer store and the selected surfaces for testing, 1-3) Payment Station, 4 & 5) Shopping Carts and Baskets, 6) Deli Counter, and 7) Refrigerated Food Section.

Sampling was performed in high-touch areas within the retailer's stores. The surfaces were selected based on their accessibility to both employees and consumers. A prior study on the transfer of viruses (other than SARS-CoV-2) from surfaces to consumers was used to select the sampling sites (Sinclair et al., 2018). Four zones within the store were selected, namely, the payment station, the deli counter, the refrigerated foods section and the carts and baskets (See Fig. 1). The payment station, carts and baskets were chosen as prior studies found a high viral and bacterial concentration on these surfaces from touch transfer (Gündüz et al., 2019; Sinclair et al., 2018). The deli counter and refrigerated food sections were chosen due to the increased traffic caused by COVID-19. The purchase of frozen and prepared foods increased, particularly early on in the pandemic (Mintel Group Ltd, 2020a, 2020b). Furthermore, the virus can remain stable at refrigeration and freezing temperatures (Duda-Chodak et al., 2020). Therefore, the deli counter and refrigerated food sections were indicated as high-touch surfaces with potential for viral contamination, persistence and transfer, and subsequently, a high risk.

The researchers collected samples at their assigned store two days per week, on Tuesdays and Fridays, for one month (mid-October to mid-November). The testing days (Tuesdays and Fridays) were selected based on current information on consumer behaviour. Tuesday has been reported to be the least preferred day for grocery shopping, and Friday and Saturday, the most preferred days (VuMa, 2020) 2020). Furthermore, as of April 2020, i.e., the beginning of pandemic lockdowns, 54% of Canadians have been reported grocery shopping at least once a week (Ledger, 2020).

Each day, samples were collected two times during the day, before starting daily operations and at the end of the working day, to evaluate the potential public's contribution to the contamination of the surfaces. The researchers arrived 1 h before opening and immediately after closing the stores. The stores' general managers were aware that sampling would take place and provided access to the store since they were closed to the general public at the time of the sampling. The personnel was informed that sampling would take place, but no details were provided on the nature of the sampling or the targeted areas. Neither the manager nor the employees supervised or intervened during swabbing. Although the sampling zones were preselected based on their potential to harbour the virus, the researchers randomly chose surfaces within these zones to be tested at each time. Different surface areas were swabbed in the morning and evening to avoid sampling areas that may have been cleaned after sampling. A total of 15 samples were taken at each location and time. The stores' cleaning, sanitation, and disinfection routines did not change or were altered due or during sampling. In the morning, the researchers arrived before the cleaning crew, and in the evenings, they performed sampling during re-stocking (prior to the final clean-up of the store). Once the store opened, a sanitation routine was conducted every 2 to 3 h. The only surfaces that received more attention, as it relates to cleaning, were the conveyor belts at the payment stations, which were sanitized before a new customer approached, and the carts and basket handles, which were sanitized before each use. This practice was implemented by the stores due to the pandemic and was already underway prior to this study.

### 2.3 Surface swabbing and testing for SARS-CoV-2

The participating researchers were trained on the use of personal protective equipment (PPE), procedures for sample labelling, collection, handling, storage, and traceability. They wore PPE (i.e., face mask and face shield) in compliance with the retailer's requirements and used gloves during sample collection (swabbing) to prevent any potential contamination of the sample. The health of all participating researchers was monitored two weeks prior to sampling, during sampling, and two weeks after.

The data collection procedure covered two stages: a) sampling of high-touch surfaces at the stores and b) testing the samples for the presence of viral RNA at the Purity-IQ laboratory (Mississauga, ON, Canada). During the sampling stage, a 25 cm^2^ (5 cm × 5 cm) area was swabbed using sterile collection swabs and transport media (PuritanTM Liquid Aimes Transport Swabs, Maine, USA). Once swabbing was performed, the samples were labelled using a code that included information on the sampling day and time, store, zone and surface tested, and the sample number. In each sampling round, a control sample (i.e., swabs that remained unused), which acted as an additional control, was also tested alongside the surface samples. The samples were stored in a cooler and transported for further evaluation. In the testing stage, the refrigerated samples (temperature ~4 °C) were transferred to the Purity-IQ Inc. laboratory for processing and detection. SARS-CoV-2 RNA detection was performed using a molecular test, i.e., reverse transcriptase quantitative polymerase chain reaction (RT-qPCR). A commercially available system that included the bCUBE® instrument with filters calibrated for FAM™ and HEX™ (Hyris, London, UK) and a reagent kit (Purity-IQ bKIT SARS-CoV-2 Environmental) was used to detect the presence or absence of SARS-CoV-2 in the samples. Health Canada has approved this system and listed it as an authorized testing device for uses related to COVID-19 (Health Canada, 2020).

Samples were run in batches of 6. Each run also included a positive and a negative control. Two repetitions of each sample and the positive and negative controls were included per run. The FAM™ emission reports on the positive control and viral targets, while the HEX™ emission reports on the negative control. Results for both positive and negative controls had to be valid in order for the test results to be validated. The reported limit of detection (LOD) of qPCR on the BCUBE, using FAM™ as a reporter dye, is 5 × 10^−6^ ng/μl (Hyris, 2018). A report of results was then generated from the bCUBE AI algorithm, and positive and negative sample results were also returned.

### 2.4 Incidence of COVID-19 in the surrounding communities

The incidence of COVID-19 in the community was recorded for the period that the study lasted. The purpose of this compilation was to establish a potential correlation between the number of cases in the respective cities/towns and the presence on surfaces within retailers. The number of cases reported at the national, provincial, and local levels was collected from sources summarized in the supplementary material (Table S1).

## 3 Results and discussion

A total of 957 samples were collected in four selected food-retail stores located in Ontario, Canada. Due to the reported survival of the SARS-CoV-2 virus on various surfaces, a range of high-touch surface areas, accessible to both employees and customers, were tested. The high-touch surface areas in retail stores were identified in 4 zones, namely the payment station, the deli counter, the refrigerated food section, and carts and baskets and on various surface types including glass and plexiglass separations, metal bumpers, plastic and metallic handles. The total number of samples, per zone and area within the store, and the SARS-CoV-2 RNA testing results are summarized in Table 1. Additional sampling details, including the material of each of the surfaces tested, and the number of samples at each day and location, are provided in Table S2.

**Table 1:** SARS-CoV-2 results compiled by store zone and tested surface.

Zone	Area Swabbed	Number of Tests	Number of Positive Outcomes
Payment Station	Debit Machine	128	0
	Plexiglas	64	0
	Conveyor Belt	128	0
Deli Counter	Glass	128	0
	Front Panel	64	0
	Upper Panel	64	0
Refrigerated Goods Section	Handles	192	0
Carts	Handle	79	0
	Front	63	0
Baskets	Handle	47	0

Regardless of the store's location, the sampling day or time, the location of the surface within the store, or the surface material, all the samples tested negative for SARS-CoV-2 RNA (i.e., values were below the detection limit of the method). It should be noted that the results for the positive and negative controls were valid (i.e., within range) for all testing dates and that the internal controls were also within adequate range for all samples. The internal controls' acceptable range was between quantification cycle (Cq) 28-31 as a higher number may indicate sample inhibition. It is important to note that the surfaces were only tested for SARS-CoV-2 RNA. The presence of other viral or bacterial contamination was not tested. However, the lack of sample inhibition during the PCR test suggests that cleaning and sanitation were not performed immediately prior to sampling.

The number of daily cases and cumulative cases observed during the testing period are presented in Supplementary Figs. S1A and S1B. The increase in the number of daily cases at all store locations tested throughout the study did not result in an increase in the presence of SARS-CoV-2 RNA on the selected surfaces. The retailers did not change their cleaning, sanitation, or disinfection routines or any other practice during the period that this study was conducted and as the number of cases increased.

These results suggest that the risk of exposure from contaminated high-touch surfaces within a food retailer store is low. This is contingent on retail stores' enforcement and implementation of social distancing measures, regular sanitizing routines, and the systematic monitoring of the store personnel's health. These findings are also in line with the observations of Colaneri et al., 2020a, Colaneri et al., 2020b that tested the presence of SARS-CoV-2 in inanimate surfaces in hospital wards, reporting only one positive result (n=32). It has been speculated by Goldman (2020) and Mondelli et al. (2020) that, under real-life conditions, the risk of transmission from fomites that have not been in recent contact with an infected carrier is limited due to the low concentration of viral particles on them, and the low transfer rates of virus from surfaces to, for example, hands (Bhardwaj and Agrawal, 2020). Transmission from fomites has been evaluated using modeling and quantitative microbial risk assessment (QMRA) studies, which estimated this risk to be 1 in 10000 (Pitol and Julian, 2020). Most recently, the CDC (2021) has also deemed this transmission path feasible, however very unlikely, emphasizing that the main transmission route is respiratory droplets from infected people. Furthermore, the transmission of the virus is unlikely if cleaning procedures and precautions are maintained due to the high susceptibility of the virus to disinfectant agents (Mondelli et al., 2020; Xiling et al., 2021; CDC, 2021). Hence, this work supports these assumptions and estimations. That being said, the number of infected people in stores may not be known. Sinclair, Fahnestock, Feliz, Patel and Perry (2018) found that the highest concentration of virus was found on the hands of grocery shoppers, which are ultimately transferred to the checkout counter, shopping carts, and the hands of the checkout clerk. However, this risk can be mitigated through adequate hand hygiene and surface disinfection that reduce contamination (Pradhan et al., 2020; Sinclair et al., 2018). With the emergence of new variants, there is a growing concern regarding infectivity and transmission. However, if there is not enough virus concentration on surfaces in the first place, there is a low likelihood of transmission and transfer. As such, the use of PPE and enhanced cleaning procedures may be required to ensure that we do not have unforeseen problems with potential variants.

These results emphasize the importance of preventive measures to reduce the probability of encountering SAR-CoV-2 on the assessed surfaces commonly found and frequently touched in retail stores. It should be noted that the setup of this study did not allow for discriminating the efficacy of each measure applied by the stores in preventing/eliminating surface contamination by the virus. The use of PPE could be deemed responsible for preventing the virus from asymptomatic or pre-symptomatic carriers from reaching the surfaces. The cleaning, sanitation, and disinfection protocols might have been the main cause of inactivation based on the high susceptibility of the virus to disinfecting agents. However, the results indicated that the concerted efforts in place effectively rendered the selected surfaces an unlikely reservoir of the virus. Further studies in real-life scenarios should be conducted to specifically attribute the reasons for these findings to one or several preventive measures. These negative results were obtained during the second wave of COVID-19 within the country and the province.

## 4 Conclusions

Due to the COVID-19 pandemic, there has been an increase in the purchase of groceries triggered by a higher number of meals prepared at home as well as panic buying. Transmission of COVID-19 through inanimate surfaces in retailer stores has been considered plausible due to the reported persistence of the virus on different surfaces and the high level of interactions that these places have, even during lockdowns. Despite this concern, all samples collected at food retailers in this study tested negative for SARS-CoV-2. These results suggest that the risk of exposure from contaminated high-touch surfaces within a food retailer store tested under real-life conditions is low if preventive measures and recommended sanitizing routines are maintained.

This study did not include areas closed to the public in general, such as behind-the-counter spaces, breakout rooms, docking, and stocking facilities. The project did not seek to validate the sanitation practices at these retailers or evaluate other sources of COVID-19 at these locations, such as person-to-person conditions. However, based on the social distancing protocols, PPE use and practices, and disinfecting procedures implemented by the retailers, these results suggest that sanitation at the target areas could be considered adequate. Furthermore, physical distancing and monitoring favorably contribute to making these spaces safe for the public.

With the emergence of new viral strains, the infectivity of the variants is unknown. However, reducing the probability of SARS-CoV-2 virus contamination on high-touch surfaces through the use of masks and sanitization procedures should not be disregarded, and policies should be supported.

## CRediT authorship contribution statement

**Maleeka Singh:** Resources, Methodology, Writing – original draft, Writing – review & editing. **Azin Sadat:** Writing – original draft, Visualization. **Reihaneh Abdi:** Investigation. **Louis A. Colaruotolo:** Investigation. **Alyssa Francavilla:** Investigation. **Katherine Petker:** Investigation. **Pedram Nasr:** Resources, Project administration. **Maryam Moraveji:** Resources, Project administration. **Gyllian Cruz:** Data curation, Resources. **Yinan Huang:** Data curation, Resources. **Aditi Arora:** Data curation. **Aleana Chao:** Resources, Data curation. **Sarah Walker:** Resources, Data curation. **Xinya Wang:** Resources, Data curation. **Sujani Rathnayake:** Writing – original draft, Resources, Data curation. **Subramanyam Ragupathy:** Formal analysis, Project administration. **Steven G. Newmaster:** Conceptualization, Methodology, Writing – review & editing, Funding acquisition. **Robert H. Hanner:** Conceptualization, Methodology, Writing – review & editing. **Lawrence D. Goodridge:** Conceptualization, Methodology, Writing – review & editing. **Maria G. Corradini:** Conceptualization, Methodology, Writing – original draft, Writing – review & editing, Project administration, Supervision, Funding acquisition.

## Declaration of competing interest

The authors declare that they have no known competing financial interests or personal relationships that could have appeared to influence the work reported in this paper.
