# Supplementary material for: Detection of SARS-CoV-2 on surfaces in food retailers in Ontario
Source: Curr Res Food Sci. 2021 Aug 31;4:598–602. doi: 10.1016/j.crfs.2021.08.009 (PMC8406517; doi:10.1016/j.crfs.2021.08.009)
Supplement: Multimedia component 1 [file mmc1.docx]

**Supplementary Material - Detection of SAR-CoV-2 on Surfaces in Food Retailers in Ontario**

**Table S1. Sources used to compile information on daily COVID-19 cases at the different segregation levels**

| **Level** | **Details** | **Source** |
| --- | --- | --- |
| National | Canada | Corona Virus Resource Center - John Hopkins Univ^1^ |
| Province | Ontario | Public Health Ontario^2^ |
| Local | Location #1 | City of Hamilton – Information on Public Health^3^ |
|  | Location #2 | Public Health Wellington-Dufferin-Guelph^4^ |
|  | Location #3 | Halton Region Services^5^ |
|  | Location #4 | Coronavirus Response Dashboard – Mississauga^6^ |

1. Corona Virus Resource Center - John Hopkins Univ. https://coronavirus.jhu.edu/map.html
2. Public Health Ontario. COVID-19: Epidemiologic summaries. https://covid-19.ontario.ca/covid-19-epidemiologic-summaries-public-health-ontario. Last accessed 25-Nov-2020
3. City of Hamilton – Information on Public Health. https://www.hamilton.ca/coronavirus/status-cases-in-hamilton. Last accessed 25-Nov-2020.
4. Public Health Wellington. Dufferin Guelph. https://www.wdgpublichealth.ca/your-health/covid-19-information-public/status-cases-wdg. Last accessed 25-Nov-2020.
5. Halton Region Services. https://www.halton.ca/For-Residents/Immunizations-Preventable-Disease/Diseases-Infections/New-Coronavirus/Status-of-COVID-19-Cases-in-Halton?viewmode=0 . Last accessed 25-Nov-2020.
6. Coronavirus Response Dashboard – Mississauga. https://coronavirus-response-mississauga-1-mississauga.hub.arcgis.com/. Last accessed 25-Nov-2020.

**Table S2. Summary of the details of the collected samples**

| **Location** | **Zone** | **Area** | **Material** |
| --- | --- | --- | --- |
| #1 | Payment Station | Debit Machine (2) | Plastic |
|  |  | Plexiglas | Plexiglas |
|  |  | Conveyor Belt (2) | Stainless Steel |
|  | Deli Counter | Glass (2) | Glass |
|  |  | Front Panel | Stainless Steel |
|  |  | Upper Panel | Stainless Steel |
|  | Refrigerated Goods Section | Steel Handles (3) | Stainless Steel |
|  | Carts | Handle | Plastic |
|  |  | Front | Steel |
|  | Basket | Handle | Plastic |
| #2 | Payment Station | Debit Machine (2) | Plastic |
|  |  | Plexiglas | Plexiglas |
|  |  | Conveyor Belt (2) | Stainless Steel |
|  | Deli Counter | Glass (2) | Glass |
|  |  | Front Panel | Stainless Steel |
|  |  | Upper Panel | Stainless Steel |
|  | Refrigerated Goods Section | Handles (2) | Stainless Steel |
|  |  | Plastic Handle | Plastic |
|  | Carts | Handle | Plastic |
|  |  | Front | Steel |
|  | Basket | Handle | Plastic |
| #3 | Payment Station | Debit Machine (2) | Plastic |
|  |  | Plexiglas | Plexiglas |
|  |  | Conveyor Belt (2) | Stainless Steel |
|  | Deli Counter | Glass (2) | Glass |
|  |  | Front Panel | Stainless Steel |
|  |  | Upper Panel | Stainless Steel |
|  | Refrigerated Goods Section | Handles (3) | Plastic |
|  | Carts | Handle (2) | Plastic |
|  |  | Front | Steel |
| #4 | Payment Station | Debit Machine (2) | Plastic |
|  |  | Plexiglas | Plexiglas |
|  |  | Conveyor Belt (2) | Stainless Steel |
|  | Deli Counter | Glass (2) | Glass |
|  |  | Front Panel | Plastic |
|  |  | Upper Panel | Vinyl/paperboard hybrid |
|  | Refrigerated Goods Section | Handles | Stainless Steel |
|  |  | Handles (2) | Plastic |
|  | Carts | Handle | Plastic |
|  |  | Front | Steel |

**Figure S1 A & B.Number of COVID-19 Cases during the Tested Period**

| **CANADA** | **ONTARIO** |
| --- | --- |
| **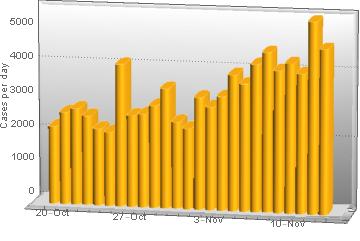** | **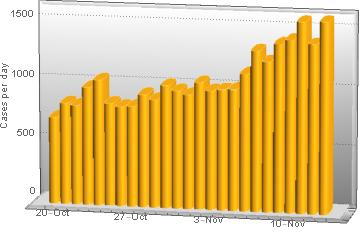** |
| **Location #1** | **Location #2** |
| 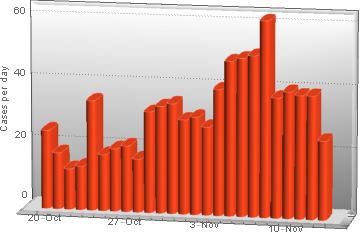 | 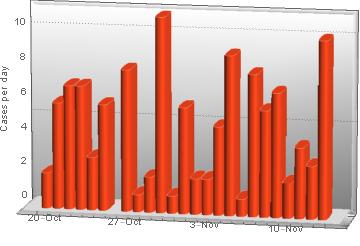 |
| **Location #3** | **Location #4** |
| 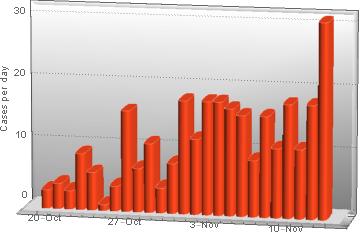 | 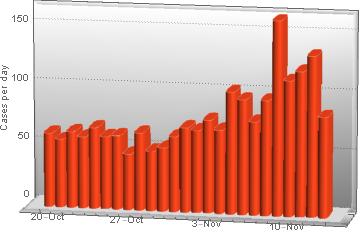 |

**Figure 1A. Number of cases reported at the national, provincial and local level during the period that testing took place (20-Oct-2020 to 13-Nov-2020). It should be noted that to facilitate observing the trend, the scale is different in all cases.**


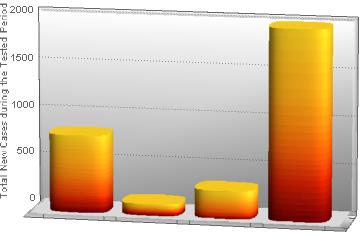


Locations

#1

#4

#2

#3

**Figure S1B. Total number of cases reported at each location during the period that testing took place (20-Oct-2020 to 13-Nov-2020)**
